# Toward an Ecological Approach to Prospective Memory? The Impact of Neisser's Seminal Talk on Prospective Memory Research

**DOI:** 10.3389/fpsyg.2019.01005

**Published:** 2019-05-03

**Authors:** Beat Meier

**Affiliations:** Institute of Psychology, University of Bern, Bern, Switzerland

**Keywords:** prospective memory, ecological validity, intention, plan, laboratory study

Prospective memory is important because it enables a person to lead an autonomous life by remembering duties, chores, and appointments. Examples such as remembering to pick up the kids at daycare on the way home from work, meet your doctor to check the blood pressure tomorrow at 2 o'clock, or remembering to take your anticoagulants are some of the typical examples.

In his seminal talk entitled “Memory: What are the important questions?” Neisser (1978) harshly criticized mainstream memory psychology because its lack of ecological validity. Neisser outlined some ecologically important questions that have resulted in a substantial research interest in the meantime. Amongst other areas such as involuntary memories, childhood memories, the function of memory for self-improvement, and eyewitness memory, Neisser emphasized that “memory is also involved in many activities of daily life. We make a plan and have to remember to carry it out.” This kind of memory has become a focus of memory research under the label “prospective memory.”

In an initial phase, prospective memory research was characterized by naturalistic research paradigms that involved calling the experimenter by telephone or sending postcards, and many of these studies can be criticized by the lack of rigorous experimental control. In order to combine the advantage of ecological valid tasks and laboratory control, naturalistic task were adopted for the use in the lab (for example, remember to sign a sheet, Dobbs and Rule, 1987; remember to hang up a telephone receiver, Kvavilashvili, 1987). Some eminent memory researchers were involved and contributed to the emerging topic by introducing theoretical distinctions or empirical observations (Loftus, 1971; Wilkins and Baddeley, 1978; Baddeley and Wilkins, 1984).

Baddeley and Wilkins (1984) introduced a distinction between different kinds of prospective memory domains, such as prospective semantic memory for describing action slips vs. prospective episodic memory “to remember an arbitrary novel action.” The latter domain has become the main focus of prospective memory research by now while the former has not received as much attention. Similarly, they distinguished between short- and long-term prospective memory, as in the domain of retrospective memory. Notably this distinction has not been integrated on a theoretical level, although it would be easy to classify “modern” paradigm according to this dimension. Overall, this initial phase of prospective memory research was characterized by many interesting observations, some theoretical distinctions, but no systematic agenda.

Ironically, a systematic investigation of the field did not begin before the topic was brought into the laboratory. A milestone was the publication of the seminal study by Einstein and McDaniel (1990). This study received a lot of attention mainly due to two reasons. First, an easy-to-use laboratory paradigm was introduced in which a prospective memory task was embedded in an ongoing task. The essential idea was to have participants busily working on one task, while at the same time requiring them to perform a different activity when a particular event occurred. From an ecological approach point of view, this situation was considered as similar to drive home from work (ongoing task) with the intention to stop at the supermarket (i.e., the target event) to buy groceries. Specifically, Einstein and McDaniel used a short-term memory task as an ongoing task which involved the presentation of different word lists. The prospective memory task consisted of remembering to press a particular key on a computer keyboard when a particular target word appeared within a short term memory test list. The word was presented repeatedly (i.e., three times) in order to achieve a more reliable measure of prospective memory. The second reason why the study attracted a lot of interest was because it provided an unexpected result. Motivated by Craik's framework of age-related memory effects, according to which tasks that require more self-initiated processes result in the strongest age-related decline, Einstein and McDaniel hypothesized that prospective memory tasks would result in large age effects due the requirement to self-initiate retrieval (Craik, 1986). Surprisingly, however, across two experiments no age effects materialized suggesting that prospective memory may differ markedly from retrospective memory. Although follow-up research has demonstrated that, in general age effects do indeed occur even in prospective memory (e.g., Zimmermann and Meier, 2006, 2010; Uttl, 2008), this study has attracted a lot of interest and has initiated a substantial body of follow-up research.

Features of the prospective memory targets such as saliency, emotional value, distinctiveness, familiarity, and specificity have been identified to influence prospective memory performance (Brandimonte and Passolunghi, 1994; Einstein et al., 1995; McDaniel et al., 1998; Pedale et al., 2017). Similarly, features of the ongoing task, in particular the cognitive resources necessary to perform the ongoing task and those still available for performing the prospective memory performance were found to influence prospective memory performance (Einstein et al., 1997; Kidder et al., 1997; Marsh and Hicks, 1998; Meier and Zimmermann, 2015). Moreover, research has identified that the interplay between those processes relevant for the prospective memory task and those relevant for performing the ongoing task also determines prospective memory task performance, with better prospective memory performance for situations with high compared to low processing overlaps (Maylor, 1996; Marsh et al., 2000; Meier and Graf, 2000). This has led to the distinction between focal and non-focal tasks, with the former being triggered spontaneously and the latter requiring demanding monitoring processes.

Accordingly, the assessment of ongoing task costs became an integral part of laboratory prospective memory research. In fact, one theory claimed that successful prospective memory performance is always accompanied by a performance cost, due to the dual task nature of the laboratory prospective memory task setting (Smith, 2003; Smith et al., 2007; Smith, 2010; see Einstein and McDaniel, 2010, for a critical commentary). In order to achieve a more sensitive measure of ongoing task costs, prospective memory tasks were embedded in time-critical decision tasks (e.g., lexical decisions) and speed of ongoing task performance without additional prospective memory task requirement was compared to speed of ongoing task performance with the additional prospective memory task requirement. The reaction time difference was taken as a measure of cost of having the additional burden of a prospective memory task. Moreover, this set-up has proved to be well-suited to map prospective memory and ongoing task processes with mathematical models which have further stimulated the discourse on theoretical foundations of prospective memory retrieval processes recently (Horn et al., 2011; Heathcote et al., 2015; Anderson et al., 2018).

Although measuring the costs of adding a prospective memory task to an ongoing task is promising from a strictly laboratory experimental perspective, from an ecological perspective to prospective memory several questions arise. First, obviously humans would hardly be able to manage the complex requirements of everyday life if each and every intention would permanently require cognitive resources. Thus, different strategies must be available in everyday life. To resolve this issue a dynamic interplay between bottom up and top down process has been proposed (McDaniel and Einstein, 2000; Scullin et al., 2013; Shelton and Scullin, 2017). In everyday life, the context can often serve as a cue for the upcoming prospective memory retrieval occasion. That is, after spontaneous retrieval of the appropriate context, a monitoring strategy may be initiated. For example, if the intention is to stop at the supermarket to buy bread on the way home, a crossing close to the supermarket can spontaneously trigger the intention and just then resources are allocated for monitoring the appropriate exit (Meier et al., 2006).

Second, and more critically, embedding the prospective memory task into a speeded decision task may affect the ecological validity of the task. This latter concern is corroborated by research that has investigated the relationship between everyday prospective memory failures and prospective memory performance as measured in the laboratory (Unsworth et al., 2012). The results indicated that everyday prospective memory was not at all correlated to prospective memory as assessed with laboratory tasks, thus adding to the questions of ecological validity of laboratory prospective memory tasks.

It may thus be helpful to reconsider some of the considerations of Neisser (1978). Despite the progress that has been made on a conceptual level, establishing the link between laboratory prospective memory research and everyday prospective memory is still a challenge. One avenue that has been under-investigated so far is the topic of habitual prospective memory. Although taking medication according to a prescription regimen is a typical prospective memory example for research proposals, neither the definitional requirements of habitual prospective memory nor their relation to the more common concept of habits is clear (see Cuttler and Graf, 2009; McDaniel et al., 2009; Meier et al., 2014, 2019). On a methodological level, new technical developments such as the availability of virtual reality environments to reproduce real-world scenarios may re-stimulate an ecological approach to prospective memory. To put it with Neisser (1978), “the challenge will be to shift from testing hypotheses for their own sake to using them as tools for exploration of reality.”

Nevertheless, to optimize prospective memory, several lessons have been learned so far. These involve the efficiency of planning for subsequent remembering (e.g., implementation intentions as a strategy adopted from motivational psychology, cf. Gollwitzer, 1999), the efficiency of specificity (e.g., to use salient retrieval cues) and the efficiency of retrieval situations with minimal distractions.

To summarize, Neisser's seminal talk has contributed to establish prospective memory as a growing research field. However, progress has mainly been achieved through controlled laboratory studies (cf. Peelen and Kastner, 2014, for a similar situation in the domain of visual search). Thus, the challenge remains to bring the insights of the research findings back to the field in order to demonstrate their generality. Last, but not least, as almost every funding application for prospective memory research emphasizes the practical value of the research, more work on establishing the link between laboratory and real-life prospective performance should be warranted.

## Author Contributions

BM has drafted and written this article.

### Conflict of Interest Statement

The author declares that the research was conducted in the absence of any commercial or financial relationships that could be construed as a potential conflict of interest.
